# Coffee with *Cordyceps militaris* and *Hericium erinaceus* Fruiting Bodies as a Source of Essential Bioactive Substances

**DOI:** 10.3390/ph17070955

**Published:** 2024-07-17

**Authors:** Katarzyna Kała, Małgorzata Cicha-Jeleń, Kamil Hnatyk, Agata Krakowska, Katarzyna Sułkowska-Ziaja, Agnieszka Szewczyk, Jan Lazur, Bożena Muszyńska

**Affiliations:** 1 Department of Medicinal Plant and Mushroom Biotechnology, Faculty of Pharmacy, Jagiellonian University Medical College, 9 Medyczna, 30-688 Kraków, Poland; malgorzata.cicha-jelen@uj.edu.pl (M.C.-J.); kamil71.hnatyk@student.uj.edu.pl (K.H.); katarzyna.sulkowska-ziaja@uj.edu.pl (K.S.-Z.); agnieszka.szewczyk@uj.edu.pl (A.S.); jan.lazur@doctoral.uj.edu.pl (J.L.); bozena.muszynska@uj.edu.pl (B.M.); 2 Department of Inorganic Chemistry and Pharmaceutical Analytics, Faculty of Pharmacy, Jagiellonian University Medical College, 9 Medyczna, 30-688 Kraków, Poland; agata.krakowska@uj.edu.pl; 3 Department of Analytical Chemistry and Biochemistry, Faculty of Materials Science and Ceramics, AGH University of Krakow, 30 Adama Mickiewicza, 30-059 Kraków, Poland

**Keywords:** medicinal mushrooms, mushroom supplements, coffee, fruiting bodies, *Hericium erinaceus*, *Cordyceps militaris*, pro-cognitive activity, ergogenic activity, health improvement

## Abstract

Drinking coffee is a daily routine for many people. Supplement manufacturers have proposed adding powdered *Cordyceps militaris*, known for its ergogenic and immunostimulating properties, and *Hericium erinaceus*, known for its nerve growth factor (NGF)-stimulating properties, to coffee. The aim of this work was to compare the bioactive substances in three types of coffee: machine-brewed, instant, and traditionally brewed, prepared with the addition of *H. erinaceus* and *C. militaris* fruiting bodies. The analysis of bioactive substances was performed using AAS and RP-HPLC methods. Among the control samples of coffee, traditionally brewed coffee was the best source of bioelements. Considering the mushroom species tested, the best additional source of Mg, Zn, Cu, Na, K, and Ca was *C. militaris*. A slightly higher Fe content was determined for *H. erinaceus*. With the addition of *C. militaris*, the amounts of 4-feruloylquinic acid (18.6 mg/200 mL) and 3,5-di-caffeoylquinic acid (3.76 mg/200 mL) also increased. In conclusion, the *C. militaris* species has been proven to be a better source of bioactive substances as a coffee additive in the daily diet. The combination of brewed coffee and the tested mushrooms seems to be the most beneficial in terms of health-promoting effects.

## 1. Introduction

Coffee is one of the most widely consumed beverages in the world. Different brewing and extraction methods are used depending on geographical location, cultural, and social conditions and habits [1,2]. The beverage reached Europe as early as the 15th century. Instant coffee was not produced until the 1930s due to Brazil’s coffee overproduction, which exceeded the demand for the raw material [3]. 

As part of a healthy, balanced diet and active lifestyle, it is recommended to consume a moderate amount of coffee, up to four cups/day (about 400 mg of caffeine) [4,5]. Green coffee beans are roasted at high temperatures, initiating Maillard reactions or chemical reactions between amino acids and carbohydrates, to form a number of unique compounds [4]. This complex beverage contains more than 1000 bioactive compounds that are responsible for its pleasant taste and aroma. Of these, the most common is caffeine (1,3,7-trimethylxanthine), a heat-stable purine alkaloid isolated in 1820 by Runge [1,2]. Caffeine is an antagonist of adenosine receptors, as it has the ability to block them, preventing adenosine molecules from attaching to them. When adenosine binds to the receptors, we start to feel tired and sleepy. Caffeine prevents this, improves cognitive abilities, and contributes to better perception [1]. It has also been shown that the risk of Alzheimer’s disease is lower in people who regularly consume caffeinated coffee than in those who do not [1,2,6]. In addition, the physiological effects of caffeine consumption include the acute elevation of blood pressure, an increased metabolic rate, and diuresis [1]. The “coffee paradox” is that caffeine raises blood pressure in the short-term, but drinking coffee regularly is associated with a lower risk of hypertension. Daily coffee consumption is also associated with a decrease in the incidence of heart attacks [2].

The chemical composition of brewed coffee depends on a number of factors, such as the beans, their processing method, the agricultural practices used (traditional or organic), storage time and conditions, roasting degree (light, medium, or dark), roasting process, coffee type available (bean, ground, or instant), and grinding and brewing method (boiled, filtered, or espresso). All these factors contribute to the fact that we never drink two cups of coffee with the same chemical composition. In recent decades, a number of coffee-based products have appeared, and drinks obtained through various extraction techniques have been introduced to the market [1].

The largest consumers of coffee are the Scandinavian countries. The coffee market is characterized by very dynamic development, and demand is constantly growing. Some changes in consumption models can be noted. The first instant coffees were dried by dispersing liquid coffee extract in hot air. However, a high temperature causes coffee to lose its natural properties. It is being displaced by other forms like lyophilized coffee, which is much more delicate and flavorful. Instant coffee is often adulterated, for example, by adding cereal coffee, chicory, or sugar beet. The specifics of the production of instant coffee mean that it often contains in its composition substances not naturally present in the raw material [7,8]. It can be said that the development of the coffee market follows several paths. There are consumers who appreciate the ease of preparing a beverage. Other consumers want the taste to be unique. On the other hand, there are baristas who specialize in making coffee from selected beans from specific plantations, which are produced and roasted artisanally. Coffee connoisseurs are able to evaluate the aroma and flavor of a freshly prepared coffee brew in terms of a number of criteria, including acidity and body [7,8].

Increasingly, coffees with various additives, including mushrooms, are available for sale. There is a noticeable synergy in the effects of medicinal mushrooms and coffee [9,10,11,12,13]. The combination of these two ingredients is interesting, both in terms of sensory experience and health-promoting properties. Manufacturers of mushroom supplements themselves suggest adding them to coffee, which may enhance its revitalizing and restorative properties, as well as reduce psycho-physical fatigue.

The selection of two mushroom species for research was scientifically justified. It is noteworthy that *Hericium erinaceus* exhibits pro-cognitive, antioxidant, antidiabetic, anticancer, anti-inflammatory, antimicrobial, antihyperglycemic, and hypolipidemic properties. It is also used in treating Parkinson’s disease, Alzheimer’s disease, or depression [6,11,14]. The mycelium of *H. erinaceus* plays a neuroprotective role by inhibiting endoplasmic reticulum stress significantly protecting against dopaminergic damage and oxidative stress in the substantia nigra which helps prevent and support the treatment of neurodegenerative diseases. *H. erinaceus* also improves cognitive impairment by modulating the gut microbiota [15]. The main active components of this species are erinacines (diterpenoids), steroids (such as erinarols, ergosterol), alkaloids (such as hericirine, fumitremorgin), polysaccharides and lactones (vitamin B_12_-*c*-lactone) [16]. *H. erinaceus* polysaccharides have an important effect on maintaining redox homeostasis in vivo—they can effectively reduce the production of reactive oxygen species, regulate the activity of antioxidases (i.e., superoxide dismutase, catalase and glutathione peroxidase), thereby mitigating oxidative stress-induced damage to tissues and organs. *H. erinaceus* also has positive effects on chronic atrophic gastritis. Oligosaccharides, terpenes, proteins, flavonoids, phenolic compounds, alkaloids, and vitamins (vitamin C and E) in *H. erinaceus* are additionally responsible for a broad spectrum of health-promoting effects [15,16]. On the other hand, *Cordyceps militaris* shows therapeutic effects on inflammation and infections, scavenges free radicals, supports liver and kidney function, boosts the immune system, relieves stress symptoms, improves skin condition, and accelerates wound healing. *C. militaris* additionally possesses anticancer, anti-aging, antimicrobial, hypolipidemic, hypoglycemic, neuroprotective, steroidogenic and fertility-enhancing activities [17]. *C. militaris* attributes its properties to numerous bioactive compounds, including cordycepin, pentostatin, carotenoids (lutein, zeaxanthin, cordyxanthins), ergothioneine, ergosterol, cordycepic acid, polysaccharides or glycoproteins. Carotenoids not only provide the orange color of this mushroom, but are potent antioxidants and perform protective functions for the skin. Polysaccharides are credited with immunomodulatory, antioxidant, anticancer and anti-inflammatory activities. Ergothioneine, phenolic compounds and selenium, present in the fruiting bodies, may also affect the antioxidant activity of the species [17,18]. Recent studies indicate that cordycepin may be effective against the SARS-CoV-2 strain that causes COVID-19. The anticancer effect of *C. militaris* is mainly due to cordycepin (3′-deoxyadenosine). An aqueous extract of *C. militaris* inhibits proliferation and induces the apoptosis of human lung cancer cells. Cordycepin also inhibits the growth of human liver cancer cells. The mechanism of cordycepin’s anticancer effect is attributed to a reduction in the production of the chemokine receptor CxCR4, which stimulates the invasion and migration of liver cancer cells [17]. Most importantly, *C. militaris* shows ergogenic and performance-enhancing effects [12,19].

The aim of the study was to obtain fruiting bodies of *H. erinaceus* and *C. militaris*, and then to determine the validity of their use as coffee additives, as well as to determine the effect of the addition of powdered fruiting bodies of both fungal species on the qualitative and quantitative composition of three types of coffee: instant coffee, ground brewed coffee, and coffee prepared from a commercially available coffee machine in public spaces. When analyzing the contents of the determined substances, it is difficult to refer to many other works, as most often the results are given as the content in dry weight of the mushroom material or, in the case of coffees, the contents in the raw material rather than the prepared solution are determined. This emphasizes the innovative value of this research, which may have practical relevance for potential consumers.

## 2. Results

When analyzing the results of the obtained analyses, it is important to consider not only the content of bioelements in the prepared coffee drinks per cup (200 mL) but also the content of the same bioelements in both species of mushrooms (mg/100 g dry weight). The contents of Mg, Zn, Cu, Fe, Na, K, and Ca were as follows for lyophilized fruiting bodies of *H. erinaceus*: 5.60, 0.115, 0.045, 0.541, 20.9, 32.1, and 7.6 mg/100 g d.w., respectively, while for *C. militaris*, they were as follows: 8.52, 0.441, 0.069, 0.287, 26.1, 40.3, and 9.04 mg/100 g d.w.

Among the control samples of coffees, traditionally brewed ground coffee was found to be the best source of all tested bioelements (Table 1).

Considering the mushroom species tested, *C. militaris* was found to be the best additional source of Mg, Cu, Na, K, and Ca in combination with traditionally brewed coffee. A slightly higher Fe content was determined with the addition of *H. erinaceus* (0.945 mg/200 mL). Among the bioelements determined, zinc is the only exception. In brewed coffee with mushroom additives, its amounts were slightly lower (1.09 mg/200 mL for *C. militaris* and 0.912 mg/200 mL for *H. erinaceus*) compared to the control (1.10 mg/200 mL). In coffee from the machine and instant coffee, the amounts of Zn increased when *C. militaris* was added. The comparison between instant coffee and machine-brewed coffee is also interesting. For almost all bioelements (except sodium), instant coffee was a better source. Regarding the mushroom additives, it should be noted that while the addition of *C. militaris* is more preferred due to its bioelement contents, the amount of bioelements also increases after adding *H. erinaceus* each time (Table 1).

The results show slight differences for marked organic compounds. It appears that when mushroom additives are used in all three types of coffee, they lead to a reduction in the contents of substances that are typical and characteristic of coffee, such as neochlorogenic acid, caffeine, chlorogenic acid, and isochlorogenic acid (Table 2).

In the case of the other two acids, 4-feruloylquinic acid and 3,5-di-caffeoylquinic acid, only in conventionally brewed coffee, additions of *C. militaris* and *H. erinaceus* resulted in an increase in their content compared to the control. For both acids, the addition of *C. militaris* was found to be more beneficial. After its addition, 18.6 mg/200 mL of 4-feruloylquinic acid and 3.76 mg/200 mL of 3,5-di-caffeoylquinic acid were determined in brewed coffee. Also interesting is the content of two substances that do not naturally occur in coffee but are components of mushrooms—lovastatin and ergosterol. In the case of lovastatin, some amounts were observed after the addition of both *C. militaris* and *H. erinaceus* species to coffee. At the same time, the trend differed from the majority of samples, with the addition of *H. erinaceus* raising the lovastatin content to a higher degree. Brewed coffee with a mushroom addition once again proved to be the best option (0.192 mg/200 mL lovastatin). As for ergosterol, certain amounts were determined only after adding the fruiting body of the *C. militaris* species to all three types of coffee, with the highest amounts determined for ground coffee brewed in the traditional manner, at 1.01 mg/200 mL of solution (Table 2). Analyzing the contents of the labeled bioactive compounds in the three types of control coffee—without the addition of mushrooms—almost all compounds were determined in the highest amount in ground coffee brewed in a traction manner. Only 4-feruloylquinic acid was determined in the highest amount in instant coffee. The content of bioactive substances also translates into the highest antioxidant potential of brewed coffee. The antioxidant potential of brewed coffee was almost twice as high compared to instant coffee and coffee prepared in a machine (Table 2).

## 3. Discussion

Medicinal mushrooms and mushroom preparations containing bioactive compounds are classified as nutraceuticals. According to the European Food Safety Agency (EFSA), they can be used as supplements due to their disease-preventing effects. The production of nutraceuticals requires a great knowledge of the functional properties of individual mushroom species. Currently, many mushroom preparations are commercially available, usually in the form of a dry extract. Coffee is an example of a popular product with medicinal mushrooms such as *Inonotus obliquus* (chaga), *Ophiocordyceps sinensis* (cordyceps), *Lentinula edodes* (shiitake), *Hericium erinaceus* (lion’s mane), or *Ganoderma lucidum* (reishi). Its consumption regulates blood pressure, stimulates mental efficiency, increases energy, and strengthens the immune system and the performance of the body [20].

According to the manufacturers of mushroom coffee, despite its many health-promoting properties, it is not recommended for everyone. In some cases, it should be limited or completely excluded from the daily diet. First of all, it should not be consumed by pregnant and breastfeeding women. Drinking coffee with mushrooms is also not advisable for children. Certain diseases such as hypertension, as well as allergies, are also contraindicated. Furthermore, this type of supplementation can interact with some medications, such as those taken for autoimmune diseases. Another group of people for whom it is not recommended to consume coffee with mushrooms are people with histamine metabolism disorders, people with gastroesophageal reflux disease, where coffee can increase discomfort, and patients with small intestine bacterial overgrowth syndrome (SIBO), where the polysaccharides found in mushrooms can cause adverse effects [8,21].

Over time, coffee transitioned from being treated primarily as a stimulant to being recognized as a beverage with health-promoting properties. Its positive effects are mainly attributed to the polyphenols it contains. Additionally, coffee beans contain carbohydrates, proteins, fats, alkaloids, diterpenes, free amino acids, melanoidins formed during the roasting process, as well as minerals—both macronutrients and micronutrients. While coffee is not usually presented as a source of bioelements, its frequent consumption can also supplement them in the body. Coffee can provide K and Mg. In addition to macronutrients, it contains varying amounts of micronutrients. These components are equally important, even though the human requirement for them is less than 100 mg/day. Micronutrients are involved in many biochemical processes in the body. Especially important are those with antioxidant properties—Zn, Cu, and Fe. They support various mechanisms and counteract the effects of oxidative stress [22]. A portion of coffee can provide up to 13.7% of the Mn requirement, up to 4.0% and 3.1% of the Zn requirement for men and women, respectively, up to 2.7% and 2.1% of the Cu requirement for men and women, respectively, and up to 0.4% and 0.6% of the Fe requirement for men and women, respectively [22].

Caffeine is not a neutral substance for the body; it has been proven to adversely affect the concentrations of electrolytes such as Mg^2+^, K^+^, and Ca^2+^ by increasing their excretion in urine [23]. Thus, it is important to develop mushroom additives that can balance these emerging fluctuations in bioelements. The amounts of the mentioned bioelements significantly increased for both mushroom additives, ranging from several times to as much as a dozen times. However, the addition of *C. militaris* provided better supplementation of Mg^2+^, K^+^, and Ca^2+^, which are needed by the human body (Table 1). Özdestan, in a study on samples of Turkish-brewed coffees, reports the average content of Mg, Mn, Zn, Na, and K as 149.18, 2.736, 2.915, 289.7, and 570.26 mg/L, respectively. The most abundant mineral in brewed coffee samples was K. The total mineral concentration (Mg + Mn + Zn + Na + K) in brewed coffee samples ranged from 879.12 to 1134.6 mg/L, with an average value of 1014.8 mg/L, which differs from the elements measured in the current experiment [24]. The average contents of Mg, Mn, Zn, Na, and K in the ground coffee samples were 2097.5, 48.80, 417.46, 846.72, and 9262.8 mg/kg, respectively [24]. It should be noted that differences exist in the study of coffee and its elemental composition due to the approach to the material and the determination of elements directly in the powdered coffee, as well as in the form of a solution. The type of water used can significantly impact the mineral content of infusions [22]. Differences in the elemental content of different coffees may also be due to the way they are brewed and the type of soil on which the coffee was grown [24,25].

Grembecka et al.’s findings report the average content of Mg, Mn, Zn, Na, K, and Ca in ground coffee samples as 2.100, 22.4, 5.3, 420, 13.690, and 841 mg/kg, respectively [25]. Their study also shows that instant coffee provides higher amounts of bioelements to the human body than ground coffee. However, it is not an important source of them in our diet, which differs from the findings of the current experiment [25]. In our study, we found that not only is brewed coffee the best source of bioelements, but also mushroom additives like *H. erinaceus*, and especially *C. militaris* fruiting bodies, significantly increase their contents (Table 1).

**Figure 1:**
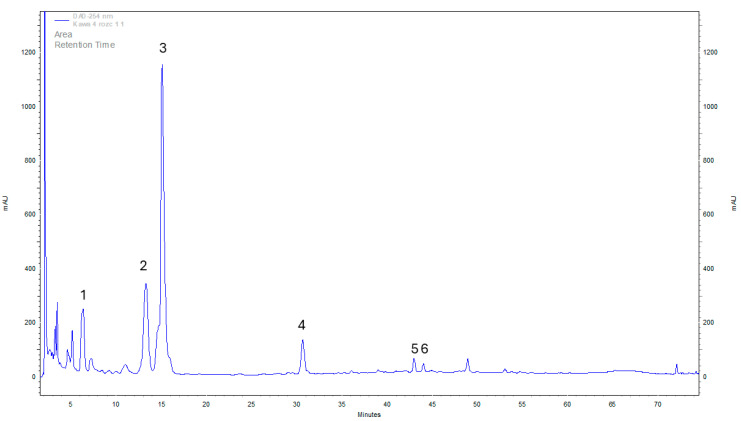
Example chromatogram of neochlorogenic acid (**1**), chlorogenic acid (**2**), caffeine (**3**), 4-feruloylquinic acid (**4**), isochlorogenic acid (**5**), and 3,5-di-caffeoylquinic acid (**6**) in the brewed coffee with *Cordyceps militaris*.

Many studies have shown that coffee is one of the most important sources of polyphenols and caffeoylquinic acids (CQAs). The main polyphenol is chlorogenic acid, which is a potent antioxidant found in coffee. About a third of the amount of chlorogenic acids ingested with coffee can be absorbed in the human digestive tract and metabolized in the stomach, intestines, liver, and kidneys, exerting a number of beneficial biological properties in the body [1,26]. Numerous meta-analyses have been performed, revealing positive health effects associated with coffee consumption, including a reduced incidence of type 2 diabetes, kidney stones, gout, Parkinson’s disease, liver fibrosis, cirrhosis, nonalcoholic steatohepatitis, and liver cancer. However, the molecular mechanism responsible for these health effects is still unresolved [2,4,6,9]. Several studies have associated CQAs with beneficial health properties, such as antioxidant, antiviral, antibacterial, anticancer, and anti-inflammatory activity [26]. This is important because substances from this group were also chromatographically identified and determined in the present experiment (Figure 1).

Coffee consumption has also been found to have a beneficial effect on the immune system, aligning with the properties of both species of mushrooms—*C. militaris* and *H. erinaceus* [10,14,19].

**Figure 2:**
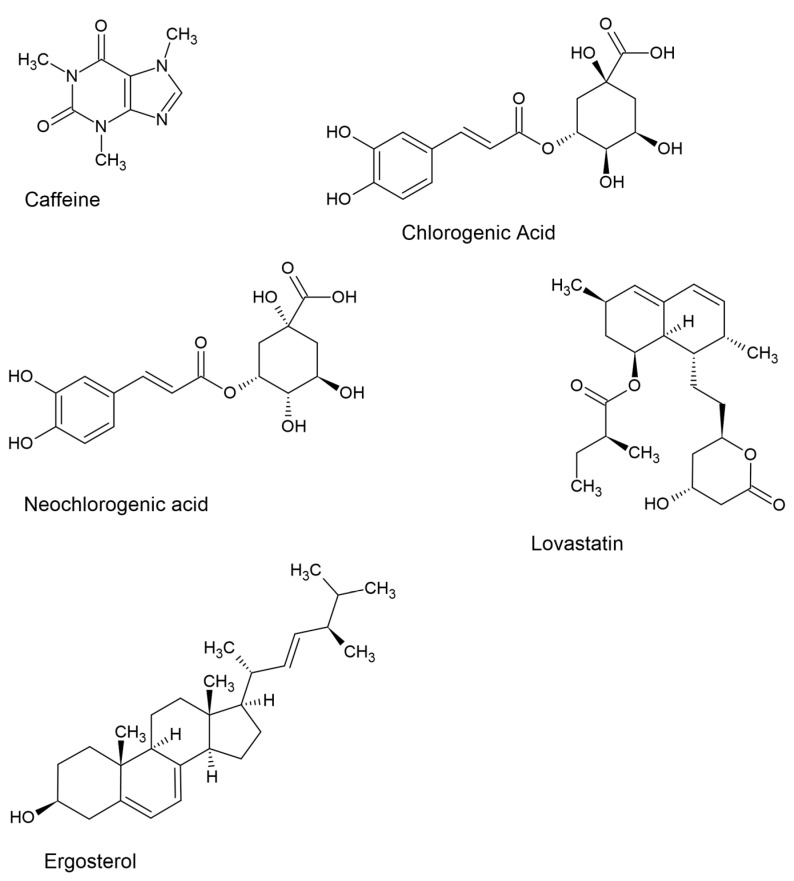
Structural formulas of bioactive substances characteristic of coffee (caffeine, chlorogenic acid, neochlorogenic acid) and mushroom material (lovastatin, ergosterol).

Analyzing the results of the organic content of the analyzed solutions, it is possible to identify substances specific to coffee itself (as mentioned above) and medicinal mushrooms (Figure 2).

Powdered fruiting bodies obtained from our own cultivation have been determined previously (Table 3) [13,27,28].

Lovastatin is one of the statins, which are inhibitors of a key enzyme regulating cholesterol production (3-hydroxy-3-methylglutaryl-coenzyme A reductase). By lowering total cholesterol and low-density lipoprotein (LDL) levels, these substances have proven effective in preventing cardiovascular diseases, including atherosclerosis, and they possess antioxidant and anti-inflammatory properties [29,30]. Mushroom sterols, including ergosterol, are converted in the presence of light into vitamin D_2_, which plays an important role in the normal functioning of the human body, cancer prevention, and immunomodulatory effects. In addition, ergosterol facilitates the absorption of bioelements such as P and Ca, contributing to normal bone formation [13,31]. It was previously observed that *H. erinaceus* is a sterol-rich species [31,32]. This observation was confirmed, as only the addition of powdered fruiting bodies of *H. erinaceus* species to coffee resulted in the appearance of ergosterol in the solutions analyzed (Table 2).

It is extremely difficult to find comparative studies where the actual content of the substances taken in with the beverage consumed (per cup) is examined. However, this time, it was decided to present the actual amounts that are taken in during the consumption of coffee supplemented with mushroom material (Table 2) [13]. 

Nevertheless, the caffeine content was lower than that previously determined by Awwad et al. for green coffee (166.72 mg/L), light roast coffee (196.35 mg/L), medium roast coffee (203.63 mg/L), and dark roast coffee (189.95 mg/L). The situation is analogous for chlorogenic acid: green coffee (543.23 mg/L), light roast coffee (270.93 mg/L), and medium roast coffee (187.45 mg/L). Only dark roast coffee (90.53 mg/L) had a lower chlorogenic acid content than traditionally brewed coffee (Table 2) [33]. Similarly, in the work of Jeon et al. (2019), higher contents of caffeine and chlorogenic acid and its derivatives were determined in both instant coffees and ready-to-drink coffee drinks, as well as in ground roasted coffee [34]. The lowest content of some substances in instant coffee in this study may be due to the production process itself and may be the result of the high temperature used to obtain instant coffee [7,8]. Concerning mushrooms, the amounts of lovastatin and ergosterol in the fruiting bodies of *C. militaris* (30.5–36.4, 95.5–142 mg/100 g d.w., respectively) and *H. erinaceus* (0.374–3.14, 139–161 mg/100 g d.w., respectively) are known. This highlights the fact that the percentages of the amounts determined in the starting extracts per dry weight will be found in the coffee drink (Table 3). In addition, a trend toward the production of higher amounts of lovastatin by *C. militaris* species and higher amounts of ergosterol for *H. erinaceus* can be observed, which translates into contents in the marked coffees [13,28].

## 4. Materials and Methods

### 4.1. Mushroom Material

The fruiting bodies of *Hericium erinaceus* (Bull.) Pers. and *Cordyceps militaris* (L.) Fr. species were obtained in cooperation with a small farm in southern Poland (Osiek, Poland). Mycelial cultures obtained from the Department of Medicinal Plant and Mushroom Biotechnology (Jagiellonian University Medical College) were used to cultivate the fruiting bodies. Stock cultures of *H. erinaceus* and *C. militaris* were obtained from The Mycelium Emporium (Enfield, ME, USA). Mushroom materials of each species were deposited into the Department of Medicinal Plant and Mushroom Biotechnology (Jagiellonian University Medical College, Kraków, Poland)—HEFB1, HEMC1, CMFB1, and CMMC1.

### 4.2. Biotechnological Methods of Obtaining Mushroom Materials

The in vitro cultures of *H. erinaceus* and *C. militaris* were obtained using a previously developed procedure and standard Oddoux media [35,36]. The composition of the culture basic medium (pH = 5.6) per 1 L was as follows: glucose 10 g, maltose extract 7.5 g, casein hydrolyzate 0.2 g, L-asparagine 1.0 g, NH_4_Cl 0.5 g, adenine 0.012 g, yeast extract 0.03 g, MgSO_4_·7H_2_O 0.5 g, KH_2_PO_4_ 0.5 g, 1% FeCl_3_ 0.6 mL, 0.3% ZnSO_4_·6 H_2_O 1.5 mL, 0.5% MnSO_4_·H_2_O 1.5 mL, and 1% CaCl_2_ 5 mL (Pol-Aura, Dywity, Poland). Increased levels of glucose, casein, and yeast extract (20, 2.5, and 0.625 g, respectively) were used to increase biomass growth in both species. The transfer of mycelial cultures was carried out in a laminar flow chamber. Shaken experimental in vitro cultures of *H. erinaceus* and *C. militaris* (3 weeks in a TOS-6048FD orbital motion rotary shaker (EnviSense, Lublin, Poland) according to the natural photoperiod) in a liquid medium were used to produce fruiting bodies of both species.

### 4.3. Obtaining Fruiting Bodies of H. erinaceus and C. militaris

To prepare the fruiting bodies of *H. erinaceus*, liquid mycelial cultures were used. They were inoculated into rehydrated (previously sterilized) wheat grains, which were then stored in Unicorn Bags—Type 3 (Plano, TX, USA) with 0.2 μm microfilters. The growing medium was prepared using a 30% wheat bran and 70% beech sawdust ratio based on volume. The substrate, totaling 2.5 kg, was rehydrated to achieve a 55% relative humidity, mixed using a stirrer, and then transferred into prepared bags. These bags were then subjected to steam sterilization (1 atm, 2 h, 121 °C). After cooling, the growing substrate was inoculated with the grained mycelium and stored in the incubation room. The prepared bags were incubated without access to light at room temperature (23 ± 2 °C). After the substrate was fully colonized, the bags were opened and transferred to a growing chamber maintained at a temperature of 20 °C with day/night-type lighting (600 lux). Two flushes of fruiting bodies were obtained, occurring after 2 and 4 weeks, respectively.

The growth medium for *C. militaris* consisted of brown rice, which is commonly used for cultivating this species. To cultivate it, 500 mL polypropylene bags were filled with 20 g of brown rice and 32 mL of a previously prepared medium. It consisted of 5 g/L peptone (BTL, Łódź, Poland), 40 g/L of glucose, 1.5 g/L of MgSO_4_·7H_2_O, and 1.5 g/L of K_2_HPO_4_ (Pol-Aura, Dywity, Poland). The composition aimed to achieve effective biomass growth and optimize yield [37]. The bags were then closed, and holes were made in the upper part, with filters glued on for proper air exchange. The polypropylene bags were then autoclaved (121 °C, 1 atm) to avoid microbial contamination. Finally, the bags with media were inoculated under a laminar cabinet with previously prepared mycelial cultures. These bags were placed in an incubation room at 20 °C for 2 weeks without light access until the entire substrate was colonized by mycelium. After mycelial growth, the bags were exposed to 600 lux of light, and the temperature was maintained at 20 °C. The first fruiting bodies appeared after 2 weeks, with harvesting conducted after 6 weeks.

Mature fruiting bodies of both species were frozen and then lyophilized at −40 °C (Labconco Freezone lyophilizer 4.5, Kansas City, MO, USA). The lyophilized fruiting bodies were powdered to prepare them for the next part of the experiments.

### 4.4. Preparation of Coffee Extracts

Three types of coffee were prepared (from a coffee machine available in public spaces (German manufacturer), instant (German manufacturer), and ground brewed (Italian manufacturer) in the traditional way) in the amount of 200 mL, both as control samples and with the addition of powdered *H. erinaceus* or *C. militaris* fruiting bodies (2 g). Traditionally brewed coffee means that the ground coffee was treated with boiling water and left to brew for a few minutes. Each coffee was obtained in three independent replicates. Two full teaspoons of instant coffee (6 g) and ground coffee (10 g) were used to prepare the drinks. The coffee from the publicly available machine was prepared automatically, with no control over the weight or type of coffee used. Water from Krakow’s waterworks, whose condition and bioelemental composition are constantly monitored, was used to make the coffee. The lyophilized *H. erinaceus* and *C. militaris* fruiting bodies were homogenized using an agate mortar. For both species, 2 g of powdered material was transferred to glass beakers containing 200 mL of previously prepared coffee. Mushrooms were added a few minutes after brewing (5 min on average). The volume of 200 mL was chosen because this is the standard volume of a cup of coffee consumed at one time. Bioelements were determined in the liquid coffee beverages. For the determination of organic bioactive substances, all types of coffee were lyophilized and then eluted to a certain volume with solvent (HPLC-grade methanol—Chempur, Gliwice, Poland) and filtered using ChemLand membrane filters (Stargard, Poland). The obtained methanolic extracts were stored in the refrigerator until further analyses. For chromatographic and spectrophotometric analyses, 2 mL of prepared extract was filtered using 0.22 µm PTFE ChemLand syringe filters (Stargard, Poland) into glass vials (Witko, Łódź, Poland).

### 4.5. Bioelement Analysis: Mineralization of Samples and Quantitative Analysis

In order to determine the contents of the selected macroelements (Ca, Mg, Na, and K) and microelements (Cu, Zn, and Fe) in the tested samples, fruiting bodies of *H. erinaceus* and *C. militaris* were subjected to mineralization. Wet mineralization was carried out in a closed system using microwaves (Magnum II mineralizer by ERTEC, Wrocław, Poland). The homogenized and lyophilized material was weighed in three independent repetitions in the amount of 0.200 g on an analytical balance with an accuracy of three places. Everything was transferred to Teflon vessels and mineralized under pressure. The clarified solution obtained in this way was transferred to quartz crucibles and evaporated at a temperature of 120 °C until almost dry. The residue was quantitatively transferred to 10 mL glass flasks using four-times-distilled water. The analysis of the content of the above-mentioned elements in the mineralizates as well as in the filtered coffee solutions was carried out using the flame atomic absorption spectroscopy (FAAS) method using the iCE3500 Thermo Scientific spectrometer (Gloucester, UK). The water used to prepare the coffee was used as a blank to eliminate measurement error. Each sample was analyzed in nine independent repetitions, and the content of bioelements was expressed in mg per 200 mL of coffee solution.

### 4.6. Analysis of the Contents of Bioactive Compounds

The chromatographic analysis of organic compounds was carried out using the HPLC method based on standard calibration curves, assuming a linear correlation between the area under the peak (AUC) and the concentration of the reference standards. Total bioactive compound content was expressed as mg per 200 mL of coffee solution. Each studied sample was analyzed in three independent replicates.

#### 4.6.1. Analysis of Caffeine and Phenolic Acids

The RP-HPLC-DAD method was used to determine the content of caffeine, chlorogenic acid, neochlorogenic acid, 4-feruloylquinic acid, isochlorogenic acid, and 3,5-di-caffeoylquinic acid. The liquid chromatography equipment (Merck Hitachi, Tokyo, Japan) consisted of an HPLC analyzer equipped with a DAD-L2455 detector, an L-2130 pump, a 4 mm × 250 mm LiChrosfer RP-18 column (particle size 5 µm), an L-2350 thermostat, and an L-2200 autosampler. For caffeine and phenolic acid analysis, we followed the gradient elution method as per Ellnain-Wojtaszek and Zgórka (1999) [38]. The injection volume was 20 µL. The mobile phase comprised two solvents: solvent A (methanol and 0.5% acetic acid in a 1:4 ratio) and solvent B (HPLC-grade methanol). The gradient profile was as follows: 100:0 from 0 to 25 min, 70:30 at 35 min, 50:50 at 45 min, 0:100 from 50 to 55 min, and back to 100:0 from 57 to 67 min (flow rate of 1 mL/min, detection at λ = 200–400 nm, column temperature maintained at 25 °C).

#### 4.6.2. Analysis of Lovastatin

The lovastatin level was determined using the RP-HPLC-UV method. The chromatography equipment (Merck Hitachi, Tokyo, Japan) used in this study consisted of an HPLC analyzer capable of isocratic separation, including an L-7400 UV detector (Merck Hitachi, Tokyo, Japan), a VWR7614 degasser, an L-7100 pump, an Purospher^®^ RP-18 4 mm × 250 mm column (particle size 5 µm), and an L-2350 thermostat. Each sample was injected with a volume of 20 µL for analysis. To determine lovastatin levels per the method reported by Pansuriya and Singhal (2009), a mobile phase consisting of acetonitrile and 0.1% phosphoric acid (60:40) was used (flow rate of 1 mL/min, detection at λ = 238 nm, column temperature set at 25 °C) [39].

#### 4.6.3. Analysis of Ergosterol

The ergosterol content was determined using the RP-HPLC-DAD method described by Yuan et al. (2008) [40]. The HPLC setup from Merck Hitachi (Tokyo, Japan) included a VWR/Hitachi LaChrom Elite analyzer with a diode-array detector (DAD L-2455), an L-2130 pump, a VWR7614 degasser, an LiChrospher RP-18 4 mm × 250 mm column (particle size 5 µm), and an L-2350 thermostat. In each instance, 20 µL of samples were injected. The mobile phase consisted of solvent A (methanol:water 8:2 *v*/*v*) and solvent B (methanol:dichloromethane 75:25 *v*/*v*). The gradient program was as follows: 100% A for 10 min, 60% A and 40% B for 10 min, 40% A and 60% B for 10 min, 20% A and 80% B for 10 min, 0% A and 100% B for 10 min, followed by reconditioning with 100% A for the next 10 min (flow rate of 1 mL/min, detection at λ = 280 nm, column temperature maintained at 30 °C).

### 4.7. Determination of Antioxidant Activity Using the DPPH^•^ Method

The antioxidant activity was determined using the DPPH radical (2,2-diphenyl-1-picrylhydrazyl) method. Each of the samples underwent the following procedure: 0.1 mL of the prepared coffee methanolic extract was dissolved in 3.1 mL of methanol and thoroughly mixed. Then, 0.1 mL of the resulting solution was mixed with 4.9 mL of 0.1 mM DPPH dissolved in methanol. The reaction mixture was shaken and incubated in the dark at room temperature for 30 min.

Following the incubation period, the absorbance of the resulting mixture was measured at 517 nm against the blank using a UV-Vis A 560 spectrophotometer (AOE Instruments Co. Ltd., Shanghai, China). The antioxidant activity was then calculated using this equation: DPPH [%] = [(A0 − A1)/A0] × 100, where A0 and A1 represent the absorbances of the reference and test solutions, respectively. To quantify the total antioxidant activity, a calibration curve of ascorbic acid was used, and the results were expressed as mg of ascorbic acid equivalent (AAE) per 200 mL of the studied sample.

### 4.8. Statistical Analysis

Each studied material underwent analysis in several independent replicates. The determination results were presented as mean values with standard deviation. A two-way ANOVA was conducted, the dependent variable was the content of the compounds, the independent variables were the addition of the mushrooms used and the type of coffee. It was performed using Statsoft STATISTICA v.14 software (Tulsa, OK, USA). The significance level was set at *p* < 0.05.

## 5. Conclusions

Adding mushroom powders to coffee is becoming an increasingly popular way of supplementation. The best source of most bioelements and organic bioactive substances is ground coffee brewed in the traditional way. Among the supplemented coffees, the best source of determined substances is traditionally brewed coffee with the addition of *C. militaris*. Traditionally brewed coffee with *H. erinaceus* has a slightly higher iron content and is notable for its ergosterol content. The combination of coffee’s antihypertensive properties (the coffee paradox) and the hypolipidemic and hypoglycemic properties of *C. militaris* may prove valuable in preventing obesity, diabetes, and the development of metabolic syndrome. In addition, it should be noted that adding mushroom material to coffee is an interesting idea that can positively affect supplementation regularity due to the large consumption of coffee in the world. In addition, the combination of the positive aspects associated with coffee consumption and the properties of both mushrooms undeniably affects the improvement of human bodily health.

## Figures and Tables

**Table 1:** Comparison between the bioelement contents of three types of coffee prepared with *H. erinaceus* and *C. militaris* with the relevant controls.

	Coffee from the Machine—Control	Coffee from the Machine with *Cordyceps militaris*	Coffee from the Machine with *Hericium erinaceus*	Instant Coffee—Control	Instant Coffee with *Cordyceps militaris*	Instant Coffee with *Hericium erinaceus*	Brewed Coffee—Control	Brewed Coffee with *Cordyceps militaris*	Brewed Coffee with *Hericium erinaceus*
mg/200 mL ± SD									
Mg	0.0397 ± 0.027 ^f^	5.68 ± 0.39 ^b,c^	4.25 ± 0.59 ^d^	1.57 ± 0.11 ^e,f^	6.69 ± 0.88 ^b^	4.59 ± 0.34 ^c,d^	1.77 ± 0.10 ^e^	8.07 ± 0.21 ^a^	5.70 ± 0.43 ^b,c^
Zn	0.196 ± 0.001 ^e^	0.338 ± 0.038 ^d^	0.153 ± 0.011 ^e^	0.565 ± 0.038 ^c^	0.590 ± 0.064 ^c^	0.253 ± 0.027 ^d,e^	1.10 ± 0.07 ^a^	1.09 ± 0.07 ^a^	0.912 ± 0.061 ^b^
Cu	0.013 ± 0.001 ^e^	0.046 ± 0.002 ^c,d^	0.040 ± 0.001 ^c,d^	0.018 ± 0.001 ^e^	0.076 ± 0.005 ^b^	0.054 ± 0.009 ^c^	0.037 ± 0.002 ^d^	0.114 ± 0.010 ^a^	0.072 ± 0.009 ^b^
Fe	0.140 ± 0.010 ^f^	0.201 ± 0.028 ^e,f^	0.424 ± 0.024 ^c,d^	0.33 ± 0.01 ^d,e^	0.274 ± 0.032 ^e,f^	0.561 ± 0.034 ^c^	0.767 ± 0.070 ^b^	0.878 ± 0.077 ^a,b^	0.945 ± 0.079 ^a^
Na	1.32 ± 0.08 ^g^	8.47 ± 0.61 ^d^	3.57 ± 0.26 ^f^	0.370 ± 0.041 ^g^	10.5 ± 0.5 ^c^	6.14 ± 0.47 ^e^	1.25 ± 0.16 ^g^	18.9 ± 0.5 ^a^	14.2 ± 0.6 ^b^
K	2.09 ± 0.14 ^f^	9.12 ± 0.56 ^c^	5.98 ± 0.28 ^d,e^	5.27 ± 0.22 ^e^	10.0 ± 0.5 ^c^	6.97 ± 0.37 ^d^	7.11 ± 0.29 ^d^	27.7 ± 1.3 ^a^	19.3 ± 0.8 ^b^
Ca	0.218 ± 0.014 ^f^	2.71 ± 0.31 ^c,d^	1.71 ± 0.25 ^e^	0.408 ± 0.033 ^f^	3.14 ± 0.29 ^b,c^	2.15 ± 0.09 ^d,e^	0.674 ± 0.032 ^f^	7.29 ± 0.52 ^a^	3.44 ± 0.23 ^b^

*n* = 9; the letters next to values represent Tukey’s HSD post hoc results, different letters ^(a–g)^ indicate significant differences between homogeneous groups (*p* < 0.05) between coffee types for each bioelement after a specific coffee additive or control.

**Table 2:** Comparison between the antioxidant activity and contents of bioactive substances of three types of coffee prepared with *H. erinaceus* and *C. militaris* with the relevant controls.

	Coffee from the Machine—Control	Coffee from the Machine with *Cordyceps militaris*	Coffee from the Machine with *Hericium erinaceus*	Instant Coffee—Control	Instant Coffee with *Cordyceps militaris*	Instant Coffee with *Hericium erinaceus*	Brewed Coffee—Control	Brewed Coffee with *Cordyceps militaris*	Brewed Coffee with *Hericium erinaceus*
mg/200 mL ± SD									
Neochlorogenic acid	9.26 ± 0.03 ^d^	6.55 ± 0.05 ^f^	4.32 ± 0.03 ^i^	8.26 ± 0.06 ^e^	4.70 ± 0.02 ^h^	4.90 ± 0.04 ^g^	17.9 ± 0.2 ^a^	10.5 ± 0.1 ^b^	10.3 ± 0.1 ^c^
Caffeine	2.71 ± 0.01 ^e^	1.77 ± 0.01 ^f^	1.15 ± 0.01 ^g^	2.97 ± 0.01 ^d^	1.74 ± 0.01 ^f^	1.72 ± 0.01 ^f^	5.59 ± 0.04 ^a^	3.76 ± 0.02 ^b^	3.62 ± 0.01 ^c^
Chlorogenic acid	9.85 ± 0.23 ^d^	6.14 ± 0.01 ^f^	4.27 ± 0.38 ^g^	4.74 ± 0.10 ^e^	4.51 ± 0.09 ^g^	4.51 ± 0.09 ^g^	33.9 ± 0.7 ^a^	21.3 ± 0.7 ^b^	19.4 ± 0.1 ^c^
4-Feruloylquinic acid	8.43 ± 0.02 ^e^	5.24 ± 0.02 ^g^	4.55± 0.01 ^h^	11.2 ± 0.1 ^c^	6.53 ± 0.03 ^f^	6.33 ± 0.03 ^f^	10.6 ± 0.1 ^d^	18.6 ± 0.1 ^a^	16.8 ± 0.1 ^b^
Isochlorogenic acid	3.20 ± 0.32 ^c^	1.44 ± 0.01 ^e^	1.56 ± 0.15 ^e^	3.63 ± 0.02 ^b^	1.39 ± 0.01 ^e^	2.02± 0.21 ^d^	5.53 ± 0.06 ^a^	3.83 ± 0.02 ^b^	3.62 ± 0.02 ^b^
3,5-Di-caffeoylquinic acid	1.79 ± 0.18 ^d^	0.947 ± 0.128 ^f^	1.03 ± 0.10 ^f^	2.79 ± 0.01 ^b^	0.972 ± 0.003 ^f^	1.37 ± 0.14 ^e^	3.53 ± 0.14 ^a^	3.76 ± 0.01 ^a^	2.48 ± 0.01 ^c^
Lovastatin	nd	0.018 ± 0.001 ^d^	0.066 ± 0.001 ^b^	nd	0.010 ± 0.001 ^e^	0.031 ± 0.001 ^c^	nd	0.011 ± 0.001 ^e^	0.192 ± 0.001 ^a^
Ergosterol	nd	0.969 ± 0.002 ^b^	nd	nd	0.336 ± 0.004 ^c^	nd	nd	1.01 ± 0.01 ^a^	nd
Ascorbic acid equivalent (AAE) mg/200 mL ± SD									
Antioxidant activity—DPPH	887 ± 34 ^cd^	937 ± 14 ^c^	670 ± 13 ^f^	882 ± 12 ^d^	868 ± 23 ^d^	746 ± 16 ^e^	1579 ± 1 ^a^	1284 ± 19 ^b^	1576 ± 4 ^a^

*n* = 3; nd—not detected; the letters next to values represent Tukey’s HSD post hoc results, different letters ^(a–g)^ indicate significant differences between homogeneous groups (*p* < 0.05) between coffee types for each compound after a specific coffee additive or control.

**Table 3:** The content of bioactive substances previously determined in the fruiting bodies of *Hericium erinaceus* and *Cordyceps militaris* species [mg/100 g d.w.].

	*Hericium erinaceus* [mg/100 g d.w.]	*Corcyceps militaris* [mg/100 g d.w.]
5-Hydroxy-L-tryptophan	91 ± 3–127 ± 5 [13]	81.1 ± 4.3 [28]
L-Tryptophan	33.0 ± 0.8–35.1 ± 2.7 [13]	6.42 ± 0.03 [28]–25.0 ± 0.3 [27]
Serotonine	nd [13]	39.4 ± 0.2 [28]
5-Methyltryptamine	3.20 ± 0.32 [13]	1.44 ± 0.01 [27]
Lovastatin	0.374 ± 0.037–3.14 ± 0.23 [13]	30.5 ± 1.5–36.4 ± 0.1 [28]
L-Phenylalanine	190 ± 3–432 ± 28 [13]	6.56 ± 0.07 [28]–366 ± 12 [27]
Ergosterol	108 ± 2–161 ± 1 [13]	95.5 ± 0.4 [28]–142 ± 3 [27]
L-Ergothioneine	207 ± 8–315 ± 12 [13]	6.47 ± 0.31 [27]
Cordycepin	not marked [13]	12.1 ± 0.1 [27]–25.9 ± 0.6 [28]
*p*-Hydroxybenzoic acid	nd [13]	0.508 ± 0.012 [27]

nd—not detected.

## Data Availability

The original contributions presented in the study are included in the article, further inquiries can be directed to the corresponding author.
